# Aspects of mental health dysfunction among survivors of childhood cancer

**DOI:** 10.1038/bjc.2015.310

**Published:** 2015-09-29

**Authors:** Miranda M Fidler, Oliver J Ziff, Sarra Wang, Joshua Cave, Pradeep Janardhanan, David L Winter, Julie Kelly, Susan Mehta, Helen Jenkinson, Clare Frobisher, Raoul C Reulen, Michael M Hawkins

**Affiliations:** 1 Department of Public Health, Epidemiology and Biostatistics, Centre for Childhood Cancer Survivor Studies, School of Health and Population Sciences, Public Health Building, University of Birmingham, Birmingham B15 2TT, UK; 2 Department of Oncology, University College London Hospital, 1st Floor Central, 250 Euston Road, London NW1 2PG, UK; 3 Department of Oncology, Birmingham Children's Hospital, NHS Foundation Trust, Steelhouse Lane, Birmingham B4 6NH, UK

**Keywords:** Mental health, childhood cancer survivors, late effects, paediatric cancer, childhood cancer, health status, quality of life

## Abstract

**Background:**

Some previous studies have reported that survivors of childhood cancer are at an increased risk of developing long-term mental health morbidity, whilst others have reported that this is not the case. Therefore, we analysed 5-year survivors of childhood cancer using the British Childhood Cancer Survivor Study (BCCSS) to determine the risks of aspects of long-term mental health dysfunction.

**Procedure:**

Within the BCCSS, 10 488 survivors completed a questionnaire that ascertained mental health-related information via 10 questions from the Short Form-36 survey. Internal analyses were conducted using multivariable logistic regression to determine risk factors for mental health dysfunction. External analyses were undertaken using direct standardisation to compare mental health dysfunction in survivors with UK norms.

**Results:**

This study has shown that overall, childhood cancer survivors had a significantly higher prevalence of mental health dysfunction for 6/10 questions analysed compared to UK norms. Central nervous system (CNS) and bone sarcoma survivors reported the greatest dysfunction, compared to expected, with significant excess dysfunction in 10 and 6 questions, respectively; the excess ranged from 4.4–22.3% in CNS survivors and 6.9–15.9% in bone sarcoma survivors. Compared to expected, excess mental health dysfunction increased with attained age; this increase was greatest for reporting ‘limitations in social activities due to health', where the excess rose from 4.5% to 12.8% in those aged 16–24 and 45+, respectively. Within the internal analyses, higher levels of educational attainment and socio-economic classification were protective against mental health dysfunction.

**Conclusions:**

Based upon the findings of this large population-based study, childhood cancer survivors report significantly higher levels of mental health dysfunction than those in the general population, where deficits were observed particularly among CNS and bone sarcoma survivors. Limitations were also observed to increase with age, and thus it is important to emphasise the need for mental health evaluation and services across the entire lifespan. There is evidence that low educational attainment and being unemployed or having never worked adversely impacts long-term mental health. These findings provide an evidence base for risk stratification and planning interventions.

Although 5-year survival from childhood cancer has risen substantially to ∼80% (Stiller, 2007), long-term survival is accompanied by an excess risk of adverse outcomes due to late effects of the cancer and its treatment. Consequently, as the number of survivors increases, it becomes ever more important to investigate the risk of such adverse effects to identify vulnerable subgroups. While previous studies have investigated health status among childhood cancer survivors, mental health sequelae remains a concern as psychological limitations or distress have been reported in both adolescent and adult survivors of childhood cancer (Schultz *et al*, 2007; Gurney *et al*, 2009; Zeltzer *et al*, 2009; Gianinazzi *et al*, 2013). Additionally, conflicting findings on mental health have been reported (Reulen *et al*, 2007; Zeltzer *et al*, 2009). By identifying survivors at risk for mental health dysfunction, appropriate monitoring and early interventions within long-term care can be undertaken through risk stratification to ensure that young people and adults achieve the best possible outcomes in terms of health and social welfare, whilst optimising the expenditure of limited resources.

The goal of this study was to investigate specific aspects of mental health dysfunction among childhood cancer survivors diagnosed between the age of 0–14 years within the British Childhood Cancer Survivor Study (BCCSS) by assessing responses to specific questions within the Short Form-36 (SF-36) survey. Although studies have assessed aspects of mental health using this survey previously (Recklitis *et al*, 2003; Pemberger *et al*, 2005; Maunsell *et al*, 2006; Reulen *et al*, 2007; Zeltzer *et al*, 2008; Zeltzer *et al*, 2009), this is the first study to our knowledge to comprehensively analyse the 10 questions comprising the role emotional, social functioning, and mental health scales, which have been shown to be the most valid among the scales as mental health measures (Ware *et al*, 1993; Ware, 2000). By looking at specific questions, one can better determine the effect of various aspects of mental health dysfunction, which may have been previously undetected in a composite score or individual scale. The potential impact of demographic, cancer, social, and economic explanatory factors on mental health were explored and external analyses comparing survivors to general population norms were conducted. In doing so, this large, population-based study provides further evidence on mental health morbidity among childhood cancer survivors, which may have important implications for clinicians, family members, and survivors with regard to minimising mental health adverse late effects.

## Materials and methods

### Study population

The BCCSS is a population-based cohort of 17 980 individuals diagnosed with cancer before the age of 15, between 1940–1991 in Great Britain, and who have survived at least 5 years (Hawkins *et al*, 2008). The cohort was ascertained through the National Registry of Childhood Tumours, which has a high estimated level of completeness (∼99%) (Kroll *et al*, 2011). Ethical approval for the study was obtained from a Multi-Centre Research Ethics Committee and every Local Research Ethics Committee in Britain (*N*=212 in total).

### Short Form-36 survey

It was important to measure both health and social impacts on quality of life to understand the effect of childhood cancer treatment on long-term mental health. To ascertain health and social outcomes, a questionnaire was sent to all survivors in the BCCSS cohort who were alive and aged at least 16 years at questionnaire send-out (questionnaire return date range: 2001–2007, questionnaire return date median: 2002). Of the 14 836 survivors who were eligible to receive the questionnaire, 10 488 (70.7%) completed the survey (Hawkins *et al*, 2008). Included in the questionnaire was the SF-36, which is a generic health survey that contains 36 questions, which measure 8 dimensions of health status. From our previous work, which studied the psychometric properties of the SF-36 in the BCCSS population, we know that this survey exhibits good validity and reliability when used in long-term survivors of childhood cancer (Reulen *et al*, 2006).

**Figure 1:**
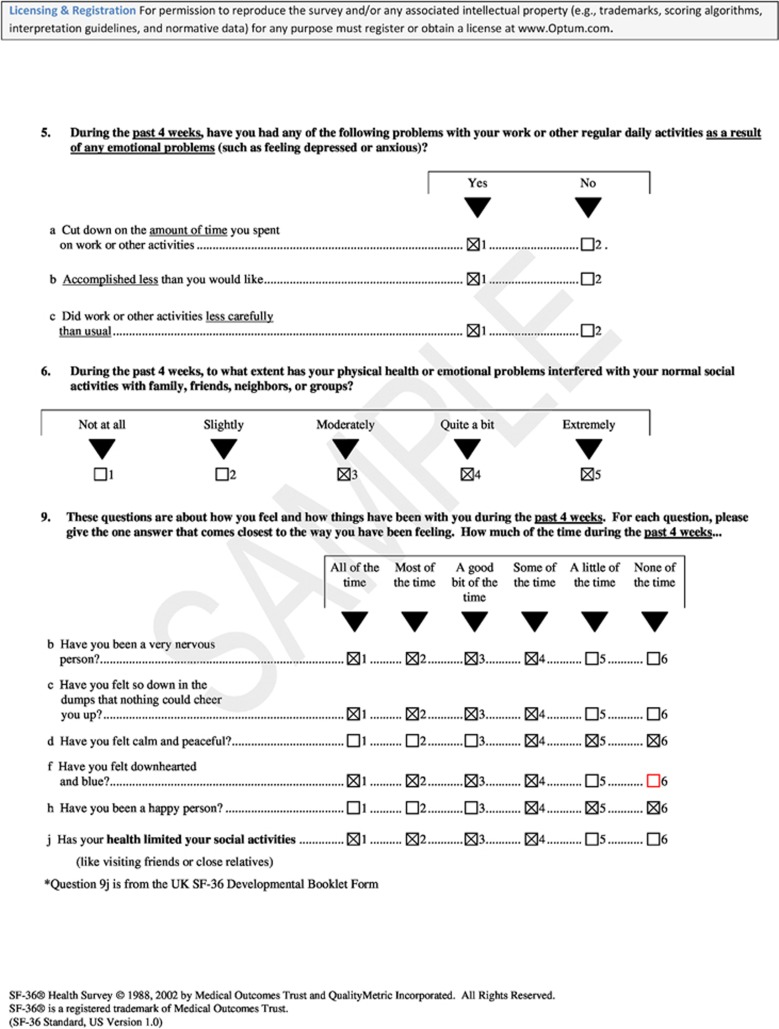
**SF-36v1 questions assessed for mental health dysfunction.** Checked boxes denote responses that were considered as ‘reporting mental health dysfunction'.

Using the available information from the SF-36, we assessed specific aspects of mental health dysfunction, henceforth only referred to as mental health dysfunction, by looking at the 10 individual questions that comprise the role emotional, social functioning, and mental health scales (Ware *et al*, 1993; Ware, 2000). To assess mental health dysfunction from the responses to each question, we dichotomised the responses (Figure 1). For the mental health scale (questions 9b, 9c, 9d, 9f, and 9h) and one question relating to social functioning (question 9j), we dichotomised the responses based on whether the sentence was positively or negatively worded, where survivors were considered to be reporting dysfunction if they answered ‘all', ‘most', ‘a good bit', or ‘some' of the time to the negatively worded questions and ‘some', ‘a little', or ‘none of the time' to the positively worded questions. The second social functioning question (question 6), which assessed physical or emotional interference in normal social activities, was dichotomised by categorising responses of ‘not at all' or ‘slightly' as not reporting dysfunction and responses of ‘moderately', ‘quite a bit', or ‘extremely' as reporting dysfunction. For the role-emotional scale (questions 5a, 5b, and 5c), survivors who reported ‘yes' were considered to be reporting mental health dysfunction. These dichotomised groupings were used to avoid the problems associated with having almost all survivors occupying one level of the dichotomy for responses to any question.

### Comparison group

To compare responses to the 10 questions between survivors and the general population, the SF-36 responses from the Oxford Healthy Life Survey (OHLS) served as the reference general population sample (Jenkinson *et al*, 1993; Jenkinson, 1996). The OHLS was conducted between 1991 and 1992 and included 13 042 individuals aged 18–64 who were randomly sampled from the Family Health Services Authority registers for Berkshire, Buckinghamshire, Northamptonshire and Oxfordshire. The OHLS sample resembles the UK general population with regard to socio-demographic characteristics (Jenkinson, 1996) and thus serves as an appropriate general population sample. Furthermore, the OHLS used identical questions and the same standardised method for self completion as the BCCSS questionnaire; when the characteristics of the OHLS were previously compared with BCCSS survivors only slight differences were observed in regards to sex and age (Reulen *et al*, 2007). The OHLS responses to the SF-36 were dichotomised as described above so that responses from survivors and the general population sample were treated identically.

### Statistical analyses–internal comparison

Internal analyses, using multivariable logistic regression, were conducted to determine risk factors for mental health dysfunction among 5-year childhood cancer survivors within each of the 10 questions. All models adjusted for the following factors: age at diagnosis, sex, first primary neoplasm (FPN) diagnosis, age at questionnaire completion, marital status, socio-economic classification, and educational attainment. We decided a *priori* to use leukaemia survivors as the referent group because previously published literature on health status has been conducted in this manner (Hudson *et al*, 2003). Odds ratios (ORs) and 95% confidence intervals (CIs) were reported. Likelihood ratio tests were used to assess the significance of fitted models and trends.

### Statistical analyses–external comparison

To compare the prevalence of mental health dysfunction between survivors and the general population, external analyses were completed using direct (age and sex) standardisation, which would address any small differences in the characteristics between the BCCSS and OHLS. For these analyses, the general population sample acted as the reference group and survivors were compared overall and separately by FPN diagnosis and attained age. Prevalence of mental health dysfunction was reported as percentages with corresponding 95% CIs.

All analyses were undertaken using Stata 13.1 (StataCorp, College Station, TX, USA). Statistical significance was defined as a two-sided *P*-value <0.05.

## Results

Survivors who were female, treated for a FPN of a central nervous system (CNS) tumour, unemployed or having never worked, or educationally unqualified were found to consistently report the highest prevalence of dysfunction across all 10 questions (Supplementary Table 1). Survivors who were separated, divorced, or widowed also generally reported more dysfunction than those who were single, cohabiting, or married. Mental health dysfunction within the 10 questions did not appear to differ substantially by age at diagnosis, treatment modalities (radiotherapy, chemotherapy, and surgery), or age at questionnaire completion.

### Internal comparison

#### Risk factors associated with reporting mental health dysfunction within the role-emotional scale

Table 1 presents the multivariable models for the three mental health questions within the role-emotional scale. Females were significantly more likely to be limited in all three questions (all *P*<0.0001). Across FPN diagnoses there were statistically significant heterogeneity for all three questions where, compared with leukaemia survivors, survivors of non-Hodgkin lymphoma (NHL), CNS tumours, and bone sarcoma were significantly more likely to be limited for all questions (all *P*<0.05). Also, compared with leukaemia survivors, heritable retinoblastoma survivors reported significantly more mental health dysfunction in having to ‘cut down on the amount of time you spent on work or other activities' (OR: 1.7(1.1–2.4)) and in having ‘accomplished less than you would like' (OR: 1.7(1.2–2.3)), whereas Hodgkin lymphoma (HL) survivors reported significantly more dysfunction in having ‘did work or other activities less carefully than usual' (OR: 1.4(1.0–1.8)). Compared with individuals aged 16–24 at questionnaire completion, the risk for reporting mental health dysfunction in all three questions increased linearly with age (all *P*trend<0.01). An analysis by marital status showed for all three questions that, relative to single survivors, those who were separated were most at risk of reporting dysfunction, whereas those who were married were significantly less likely to report dysfunction. An association was found for educational attainment, where increased qualifications were associated with decreased odds of reporting dysfunction in all three questions. For all three questions, relative to students, survivors who had never worked or were unemployed were significantly more likely to report mental health dysfunction, whereas those who were in managerial or professional positions were significantly less likely to report dysfunction.

#### Risk factors associated with reporting mental health dysfunction within the social functioning scale

In the multivariable models assessing the two questions within the social functioning scale, females were again significantly more likely to report dysfunction compared to males (Table 2). An analysis by FPN diagnosis showed that compared with those diagnosed with leukaemia, CNS (OR: 1.6(1.4–1.9)), neuroblastoma (OR: 1.5(1.1–2.0)), bone sarcoma (OR: 2.0(1.5–2.7)), and soft tissue sarcoma (OR: 1.3(1.0–1.7)) survivors were all significantly more likely to report mental health dysfunction in ‘has your physical health or emotional problems interfered with your normal social activities.' NHL, CNS, neuroblastoma, heritable retinoblastoma, bone sarcoma, and soft tissue sarcoma survivors also reported significantly higher dysfunction in ‘has your health limited your social activities', compared with leukaemia survivors, with bone sarcoma (OR: 3.0(2.3–4.0)) and CNS survivors (OR: 2.5(2.1–2.9)) being the most limited. Age at questionnaire completion was significantly associated with reporting dysfunction in both questions where those aged 25–34, 35–44, and 45+ reported more dysfunction compared with those aged 16–24 (both *P*trend<0.0001). Relative to single survivors, married survivors were significantly less likely to report dysfunction in either question (both *P*<0.001); no significant difference was found between single survivors and those who were cohabiting, separated, divorced, or widowed. An analysis by educational attainment showed that, compared with survivors with no qualifications, the odds of reporting mental health dysfunction decreased with higher levels of qualifications for both questions. Socio-economic classification was also found to be significantly related to reporting dysfunction in both questions where, compared with students, those who never worked or were unemployed were significantly more likely to report dysfunction (both *P*<0.001) and those in managerial or professional positions were significantly less likely to report dysfunction (both *P*⩽0.001).

#### Risk factors associated with reporting mental health dysfunction within the mental health scale

Survivors who were female or who had never worked or were unemployed were significantly more likely to report mental health dysfunction in all five questions within in the mental health scale relative to males and students, respectively, (Table 3). Conversely, survivors who achieved an O-level, A-level, teaching qualification, or degree were significantly less likely to report dysfunction in all questions compared with students. Age at questionnaire completion was also found to be significantly associated with reporting dysfunction in 4/5 questions, but a consistent trend was not observed within the subgroups compared with those aged 16–24. When analysed by marital status, survivors who were married were found to report significantly less mental health dysfunction in 4/5 of the questions, compared with those who were single. Survivors who were cohabiting also reported significantly less mental health dysfunction for the question relating to having ‘been a very nervous person' (OR: 0.8(0.7–1.0)). Survivors who were separated, conversely, reported a 70% increase in mental health dysfunction compared with single survivors (OR: 1.7(1.2–2.4)). When asked if the survivor had ‘been a very nervous person', those diagnosed with CNS (OR: 1.3(1.1–1.5)), compared with leukaemia, were significantly more likely to agree with this statement. Furthermore, in the multivariable model assessing whether survivors had ‘been a happy person', an analysis by FPN diagnosis showed that HL (OR: 1.3(1.0–1.6)), NHL (OR: 1.4(1.1–1.8)), CNS (OR: 1.4(1.2–1.6)), neuroblastoma (OR: 1.3(1.0–1.7)), non-heritable retinoblastoma (OR: 1.4(1.0–1.8)), and bone sarcoma (OR: 1.5(1.1–1.9)) survivors reported significantly higher dysfunction compared with leukaemia survivors.

### External comparison

Compared with the general population sample, survivors overall reported more mental health dysfunction in 6/10 questions that were examined (Table 4). When further assessed by FPN diagnosis, CNS and bone sarcoma survivors were found to report the greatest dysfunction, compared with that expected, with significant differences in 10 and 6 questions, respectively; the excess of dysfunction ranged from 4.4–22.3% in CNS survivors, whereas bone sarcoma survivors were limited from 6.9–15.9%. Both diagnostic groups were most disadvantaged by their health limiting their social activities. Conversely, survivors of neuroblastoma, heritable retinoblastoma, non-heritable retinoblastoma, Wilms, and other (those that did not conform to one of the 10 FPN groups used) were not significantly different in any of the questions analysed when compared with the general population sample.

An analysis by age at questionnaire completion showed that the prevalence of mental health dysfunction was comparable or better for 7/10 questions among survivors aged 16–24, compared with that expected from the general population sample (Table 5); survivors in this age group did however report a higher prevalence of having ‘been a nervous person' (30.7% *vs* 23.7% expected), ‘felt so down in the dumps that nothing could cheer you up' (25.3% *vs* 21.2% expected), and in ‘has your health limited your social activities' (15.7% *vs* 11.2% expected). Among those aged 25–34 and 35–44 at questionnaire completion, significantly higher mental health dysfunction was reported, compared with the general population sample, in relation to six questions (questions 5a, 6, 9b, 9c, 9f, and 9j). Similarly, survivors aged 45 and older reported significantly higher dysfunction in five questions compared with that expected. Notably, the per cent difference in mental health dysfunction between survivors and the general population increased with age at questionnaire completion for both questions from the social functioning scale and the question ‘have you felt downhearted and blue' this increase was most noticeable in the question ‘has your health limited your social activities,' where the excess rose from 4.5% to 12.8% in those aged 16–24 and 45+, respectively. Statistically significant variation in the excess by age at questionnaire completion was not observed in four questions, which related to problems with work or daily activities and feeling calm, peaceful, or happy.

## Discussion

The findings from this large population-based study indicate that the prevalence of mental health dysfunction among survivors of childhood cancer in the BCCSS was substantially higher than that expected from the general population sample in over half of the questions assessed, with survivors of CNS and bone sarcoma being the most vulnerable; these findings are generally consistent with other studies that have used the SF-36 (Maunsell *et al*, 2006) or similar psychological measures (Hudson *et al*, 2003; Zebrack *et al*, 2004; Zeltzer *et al*, 2009), although some studies have suggested that mental health status was similar between survivors and comparative populations (Reulen *et al*, 2007; Zeltzer *et al*, 2009; Phipps *et al*, 2014). While the North American Childhood Cancer Survivor Study (CCSS) found significantly higher limitations in the role emotional and social functioning scales for survivors overall, survivors of CNS and bone sarcoma were reported as having significantly less problems on the mental health scale compared to that expected from the US population reference (Zeltzer *et al*, 2008); this finding does not correspond with our results as CNS and bone sarcoma survivors were found to be significantly more limited in 5/5 and 3/5 of the questions that comprise the mental health scale, respectively. The same study (Zeltzer *et al*, 2008) also reported significantly higher limitations in regards to the role emotional and social functioning scales for HL, NHL, Wilms, and neuroblastoma survivors, compared with US norms, which conflicts with the results presented in this study as these survivors were not significantly more limited in any of the questions comprising these scales compared with the general population sample. Although the CCSS and this study used different methodologies, with the CCSS using means and this study using proportions when assessing differences between childhood cancer survivors and general population norms, broad patterns of agreement in the findings from the CCSS and this study are expected as both studies adjusted for sex and age. These inconsistencies with our study might reflect differences in study demographics, cohort design, or therapeutic practice between North America and Great Britain.

Another important finding in this study was that, although younger survivors (16–24 years) perceived their mental health as broadly similar to the general population, significant mental health dysfunction was reported in at least half of the questions among those aged 25 years and older. A particular concern was found among the questions relating to social functioning as significant mental health dysfunction was reported for all age groups. Furthermore, the extent of the excess among survivors increased with age at questionnaire completion for both questions within the social functioning scale. This finding corresponds with another study that reported significantly more disadvantage in the social functioning scale in those assessed 10–14 and 15–19 years from diagnosis compared to a control group (Maunsell *et al*, 2006). A possible explanation as to why mental health dysfunction increased with age may be due to the fact that the risk of complex and multiple late effects emerging increases as time since treatment increases (Oeffinger *et al*, 2006; Geenen *et al*, 2007; Hudson *et al*, 2013; Armstrong *et al*, 2014). Although late effects may not immediately affect survivors, they may become more important with maturity and influence life decisions and experiences later on. For example, infertility may become a greater concern and impact mental health when survivors want to start a family. Living with chronic health conditions, such as infertility, cardiovascular disease, diabetes, blindness, physical disability, and epilepsy, which can often be managed but not cured, may have long-term consequences on both physical and psychological health, stressing the importance for life course care and services.

The internal analyses similarly revealed that CNS and bone sarcoma survivors reported higher levels of mental health dysfunction compared to other types of childhood cancer, with CNS survivors being limited in all questions assessed and bone sarcoma survivors being limited in all questions relating to the social functioning and role-emotional scales. Broadly, this finding conflicts with an analysis by the CCSS, which found no significant difference among childhood cancer survivors by FPN diagnosis (Hudson *et al*, 2003); however, it is worth noting that in the CCSS the Brief Symptom Inventory 18 survey was used and thus results are not directly comparable. Other risk factors for mental health dysfunction included being female, separated from a spouse/partner, and unemployed or having never worked, which corresponds with previous reports using the SF-36 (Zeltzer *et al*, 2008; Zeltzer *et al*, 2009) or similar measures to predict psychological distress (Hudson *et al*, 2003). Low educational attainment, unemployment, and other socio-economic disadvantages are recognised risks to mental health in the general population (Marmot, 2010). However, the effects of these determinants may be even more detrimental among childhood cancer survivors as these individuals, when assessed with comparative norms, experience an even greater risk of morbidity and adverse psychosocial outcomes (Mitby *et al*, 2003; de Boer *et al*, 2006; Oeffinger *et al*, 2006; Frobisher *et al*, 2007; Geenen *et al*, 2007; Pang *et al*, 2008; Lancashire *et al*, 2010; Hudson *et al*, 2013; Armstrong *et al*, 2014). Conversely, survivors who received some educational qualifications or worked in a managerial/professional position were found to exhibit less mental health dysfunction compared with their respective referents, which also generally corresponds with previous reports (Zeltzer *et al*, 2008).

### Limitations

Response bias due to selective responses should be minimal due to our reasonably good response rate and the fact that there was not a substantial difference in cancer and socio-demographic characteristics between responders and non-responders of our questionnaire (Reulen *et al*, 2007). There is potentially selection bias due to survival, particularly among the group of older survivors as they may be healthier than their counterparts who did not survive until questionnaire send-out. Another limitation in our study is our comparison data, which may differ from our study population in terms of socio-economic status. However, as the results from our internal and external analyses broadly correspond with one another, confounding by this factor should be limited. Another limitation of this study is the lack of detailed treatment information, which precluded any analyses being completed by clinically relevant radiotherapy, chemotherapy, or surgery categories. However, in our analyses we have included FPN diagnosis, which can serve as a proxy for treatment and still offer meaningful clinical importance. In addition, this study has only assessed self-reported mental health dysfunction. Although we do not identify the risk of extreme mental health impairment (i.e., through psychiatric admissions in hospitalisation registries), the purpose of this study was to quantify daily mental health morbidities for all individuals included in the study and assess in more detail the degree of limitation in specific questions of mental health. Finally, although the findings from this paper may not be generalisable for children diagnosed with cancer after 1991, they are still highly relevant to children treated more recently for whom treatment intensity and long-term morbidity may be greater. We acknowledge reassessment is necessary and recommend further analyses to be conducted on the recently extended BCCSS cohort, which includes 5-year survivors diagnosed from 1992–2006, and other long-term follow-up studies.

### Clinical recommendations

Although the need for long-term psychological assessment and care is recognised (Chang, 1991; Eiser *et al*, 2000; Wallace *et al*, 2001; Zeltzer *et al*, 2009), there remain uncertainties as to how these individuals should be assessed. A previous study reported that approximately only 35% of childhood cancer survivors in the UK were on hospital follow-up (Taylor *et al*, 2004). Consequently, as general practitioners provide health care for the majority of these survivors (Oeffinger, 2004), routine psychological assessment, preferably using a standardised and validated measure, should be integrated into both long-term hospital follow-up clinics and general practitioner visits, especially for the vulnerable subgroup of survivors identified in this study. To date, psychological provisions are lacking in late effects services and are rare in the primary care setting. To improve mental health, it is essential that recommendations for risk-based care are readily available for general practitioners and ongoing communication is coordinated across all sites and services involved. Furthermore, surveillance for mental health dysfunction and recommended interventions should be included in the development of clinical guidelines, treatment summaries, and patient care plans. As the results from this study suggest mental health dysfunction is a concern across the lifespan for survivors, it is imperative that equitable psychological support is continuously available within general practices or specialist late effects services, irrespective of the amount of time that has passed since initial diagnosis, and that funding is allocated to allow for interventions. Finally, the findings presented in this study also stress the importance of educational attainment and employment on long-term mental health. Educational support and career advisors should be provided during and after treatment to ensure that childhood cancer survivors achieve their full educational and employment potential and have the same likelihood of academic and professional success as their peers. By continually improving the standard of care for mental health in childhood cancer survivors, we work towards meeting the goal of psychosocial oncology research, which is to facilitate patients' adjustment to the short- and long-term consequences of their treatment, recovery, and survivorship so that quality of life is not reduced (Holland *et al*, 1998).

## Conclusion

Based upon the findings of this large population-based study, childhood cancer survivors report significantly higher levels of mental health dysfunction than those in the general population, where excesses were observed particularly among CNS and bone sarcoma survivors. Limitations were also observed to increase with age, and thus it is important to emphasise the need for mental health evaluation and services across the entire lifespan. There is evidence that low educational attainment and being unemployed or having never worked adversely impacts long-term mental health. These findings provide an evidence base for risk stratification and planning interventions.

## Figures and Tables

**Table 1:** Multivariable logistic regression models^a^ reporting ORs and 95% CIs for reporting mental health dysfunction within the three questions^b^ comprising the role-emotional scale of the SF-36 survey, by specific potential demographic, cancer, social, and economic factors

	**Cut down on the amount of time you spent on work or other activities? (question 5a)**			**Accomplished less than you would like? (question 5b)**			**Did work or other activities less carefully than ususal? (question 5c)**		
**Characteristic**	**OR (95% CI)**	***P*****-value**	***P*****heterogeneity**^c^ (***P*****trend**^d^)	**OR (95% CI)**	***P*****-value**	***P*****heterogeneity**^c^ (***P*****trend**^d^)	**OR (95% CI)**	***P*****-value**	***P*****heterogeneity**^c^ (***P*****trend**^d^)
**Sex**									
Male	1.0			1.0			1.0		
Female	1.6 (1.4–1.8)	<0.001	<0.0001	1.5 (1.3–1.7)	<0.001	<0.0001	1.8 (1.6–2.0)	<0.001	<0.0001
**Diagnosis**									
Leukaemia	1.0			1.0			1.0		
Hodgkin lymphoma	1.2 (0.9–1.5)	0.317		1.0 (0.8–1.3)	0.723		1.4 (1.0–1.8)	0.024	
Non-Hodgkin lymphoma	1.4 (1.1–1.9)	0.019		1.4 (1.1–1.7)	0.019		1.6 (1.2–2.1)	0.001	
Central nervous system	1.6 (1.4–2.0)	<0.001		1.5 (1.2–1.7)	<0.001		1.5 (1.2–1.8)	<0.001	
Neuroblastoma	1.4 (1.0–1.9)	0.055		1.3 (1.0–1.7)	0.077		1.1 (0.8–1.6)	0.437	
Non-heritable Retinoblastoma	1.1 (0.8–1.6)	0.496		1.1 (0.8–1.5)	0.687		1.0 (0.7–1.4)	0.967	
Heritable retinoblastoma	1.7 (1.1–2.4)	0.011		1.7 (1.2–2.3)	0.002		1.3 (0.9–1.9)	0.205	
Wilms	1.2 (1.0–1.6)	0.105		1.0 (0.8–1.3)	0.838		1.1 (0.9–1.5)	0.256	
Bone sarcoma	1.7 (1.2–2.4)	0.001		1.4 (1.1–1.9)	0.014		1.5 (1.1–2.1)	0.008	
Soft tissue sarcoma	1.5 (1.1–1.9)	0.006		1.2 (1.0–1.5)	0.082		1.5 (1.1–1.9)	0.003	
Other	0.9 (0.7–1.2)	0.673	<0.0001	1.0 (0.8–1.3)	0.693	<0.0001	1.0 (0.8–1.3)	0.927	0.0005
**Age at diagnosis**									
0–4 years	1.0			1.0			1.0		
5–9 years	1.2 (1.0–1.4)	0.029		1.3 (1.1–1.5)	0.001		1.1 (0.9–1.2)	0.530	
10–14 years	1.2 (1.0–1.5)	0.026	0.0462 (0.0282)	1.3 (1.1–1.5)	0.003	0.0019 (0.0029)	1.1 (1.0–1.4)	0.147	0.3433 (0.1380)
**Age at questionnaire completion**									
16–24 years	1.0			1.0			1.0		
25–34 years	1.4 (1.1–1.7)	0.001		1.3 (1.1–1.5)	0.001		1.3 (1.1–1.5)	0.009	
35–44 years	1.6 (1.2–2.0)	<0.001		1.6 (1.3–2.0)	<0.001		1.4 (1.2–1.8)	0.001	
45+ years	1.4 (1.1–1.9)	0.012	0.0016 (0.0004)	1.6 (1.2–2.0)	<0.001	<0.0001 (<0.0001)	1.2 (0.9–1.6)	0.124	0.0087 (0.0054)
**Marital status**									
Single	1.0			1.0			1.0		
Cohabiting	1.1 (0.9–1.4)	0.226		1.1 (0.9–1.4)	0.166		1.0 (0.8–1.2)	0.971	
Married	0.7 (0.6–0.9)	0.002		0.7 (0.6–0.8)	<0.001		0.7 (0.5–0.8)	<0.001	
Separated	1.5 (1.0–2.4)	0.047		1.6 (1.1–2.4)	0.013		1.6 (1.0–2.3)	0.031	
Divorced	1.2 (0.9–1.6)	0.333		1.1 (0.8–1.4)	0.495		1.1 (0.8–1.4)	0.613	
Widowed	0.7 (0.2–2.2)	0.603	0.0001	0.8 (0.3–2.0)	0.583	<0.0001	1.1 (0.4–2.9)	0.817	<0.0001
**Educational attainment**									
No qualifications	1.0			1.0			1.0		
Other qualifications	0.7 (0.6–0.9)	0.010		0.8 (0.6–0.9)	0.010		0.8 (0.6–1.0)	0.040	
O-level^e^	0.7 (0.5–0.8)	<0.001		0.7 (0.6–0.8)	<0.001		0.8 (0.6–0.9)	0.005	
A-level^f^	0.7 (0.6–0.9)	0.001		0.7 (0.6–0.9)	0.002		0.7 (0.6–0.9)	0.003	
Teaching qualification	0.6 (0.5–0.8)	0.002		0.7 (0.5–0.8)	0.001		0.8 (0.6–1.0)	0.037	
Degree	0.6 (0.5–0.8)	<0.001	0.0004	0.7 (0.6–0.9)	0.003	0.0044	0.7 (0.5–0.9)	0.002	0.0030
**SEC**									
Student	1.0			1.0			1.0		
Never worked/unemployed	1.5 (1.2–2.0)	0.003		1.4 (1.1–1.8)	0.003		1.3 (1.0–1.8)	0.030	
Routine/manual	0.9 (0.7–1.1)	0.424		0.8 (0.7–1.0)	0.030		1.0 (0.8–1.2)	0.893	
Intermediate	0.9 (0.7–1.1)	0.290		0.8 (0.7–1.0)	0.060		0.8 (0.7–1.1)	0.155	
Managerial/professional	0.7 (0.5–0.9)	0.005	<0.0001	0.7 (0.5–0.8)	<0.001	<0.0001	0.8 (0.6**–**1.0)	0.025	0.0004

Abbreviations: CI=confidence intervals; OR=odds ratio; SEC=socio-economic classification.

a Adjusted for age at diagnosis, diagnosis, sex, age at questionnaire completion, marital status, educational attainment, and SEC.

b To view full questions, please refer to Figure 1.

c The Pheterogeneity (two-sided) is from the likelihood ratio test for heterogeneity in the probability of reporting mental health dysfunction within this specific question, across different levels of the specified explanatory factor with adjustment for all other factors in the multivariable model. The threshold for statistical significant was 0.05.

d The *P*trend (two-sided) is from the test for trend, where the threshold for statistical significant was 0.05.

e Degree received at age 16.

f Degree received at age 18.

**Table 2:** Multivariable logistic regression models^a^ reporting ORs and 95% CIs for reporting mental health dysfunction within the two questions comprising the social functioning^b^ scale of the SF-36 survey, by specific potential demographic, cancer, social, and economic factors

	**Has your physical health or emotional problems interfered with your normal social activities? (question 6)**			**Has your health limited your social activities? (question 9j)**		
**Characteristic**	**OR (95% CI)**	***P*****-value**	***P*****heterogeneity**^c^ (***P*****trend**^d^)	**OR (95% CI)**	***P*****-value**	***P*****heterogeneity**^c^ (***P*****trend**^d^)
**Sex**						
Male	1.0			1.0		
Female	1.5 (1.3–1.7)	<0.001	<0.0001	1.5 (1.4–1.7)	<0.001	<0.0001
**Diagnosis**						
Leukaemia	1.0			1.0		
Hodgkin lymphoma	0.9 (0.7–1.2)	0.703		1.2 (0.9–1.5)	0.284	
Non**-**Hodgkin lymphoma	1.3 (1.0–1.7)	0.079		1.5 (1.1–2.0)	0.004	
Central nervous system	1.6 (1.4–1.9)	<0.001		2.5 (2.1–2.9)	<0.001	
Neuroblastoma	1.5 (1.1–2.0)	0.011		1.5 (1.1–2.0)	0.020	
Non**-**heritable retinoblastoma	1.0 (0.7–1.4)	0.812		1.0 (0.7–1.5)	0.864	
Heritable retinoblastoma	1.3 (0.9–1.9)	0.197		1.5 (1.0–2.2)	0.037	
Wilms	1.1 (0.8–1.3)	0.896		1.1 (0.9–1.5)	0.275	
Bone sarcoma	2.0 (1.5–2.7)	<0.001		3.0 (2.3–4.0)	<0.001	
Soft tissue sarcoma	1.3 (1.0–1.7)	0.036		1.6 (1.2–2.0)	<0.001	
Other	1.0 (0.7–1.2)	0.692	<0.0001	1.2 (0.9–1.5)	0.224	<0.0001
**Age at diagnosis**						
0–4 years	1.0			1.0		
5–9 years	1.1 (0.9–1.3)	0.377		1.1 (0.9–1.2)	0.462	
10–14 years	1.1 (1.0–1.4)	0.137	0.3297 (0.1368)	1.1 (0.9–1.3)	0.258	0.5227 (0.2821)
**Age at questionnaire completion**						
16–24 years	1.0			1.0		
25–34 years	1.5 (1.3–1.8)	<0.001		1.8 (1.5–2.2)	<0.001	
35–44 years	1.6 (1.3–1.8)	<0.001		2.0 (1.6–2.5)	<0.001	
45+ years	1.5 (1.2–2.0)	0.002	<0.0001 (<0.0001)	2.1 (1.6–2.7)	<0.001	<0.0001 (<0.0001)
**Marital status**						
Single	1.0			1.0		
Cohabiting	1.1 (0.9–1.3)	0.419		1.0 (0.8–1.2)	0.940	
Married	0.7 (0.6–0.9)	<0.001		0.7 (0.6–0.8)	<0.001	
Separated	1.3 (0.9–2.0)	0.176		1.2 (0.8–1.8)	0.447	
Divorced	1.1 (0.9–1.5)	0.339		1.0 (0.8–1.3)	0.969	
Widowed	1.2 (0.5–2.9)	0.709	0.0001	1.6 (0.7–3.8)	0.255	0.0001
**Educational attainment**						
No qualifications	1.0			1.0		
Other qualifications	0.7 (0.6–0.9)	<0.001		0.7 (0.6–0.8)	<0.001	
O**-**level^e^	0.6 (0.5–0.7)	<0.001		0.5 (0.4–0.6)	<0.001	
A**-**level^f^	0.6 (0.5–0.7)	<0.001		0.4 (0.4–0.5)	<0.001	
Teaching qualification	0.5 (0.4–0.7)	<0.001		0.5 (0.4–0.6)	<0.001	
Degree	0.5 (0.4–0.6)	<0.001	<0.0001	0.4 (0.3–0.5)	<0.001	<0.0001
**SEC**						
Student	1.0			1.0		
Never worked/unemployed	1.6 (1.3–2.1)	<0.001		1.7 (1.3–2.2)	<0.001	
Routine/manual	0.9 (0.7–1.1)	0.384		0.9 (0.7–1.1)	0.402	
Intermediate	0.9 (0.7–1.1)	0.329		0.9 (0.7–1.1)	0.224	
Managerial/professional	0.7 (0.5–0.9)	0.001	<0.0001	0.7 (0.5–0.9)	<0.001	<0.0001

Abbreviations: CI=confidence intervals; OR=odds ratio; SEC=socio-economic classification.

a Adjusted for age at diagnosis, diagnosis, sex, age at questionnaire completion, marital status, educational attainment, and SEC.

b To view full questions, please refer to Figure 1.

c The Pheterogeneity (two-sided) is from the likelihood ratio test for heterogeneity in the probability of reporting mental health dysfunction within this specific question, across different levels of the specified explanatory factor with adjustment for all other factors in the multivariable model. The threshold for statistical significant was 0.05.

d The *P*trend (two-sided) is from the test for trend, where the threshold for statistical significant was 0.05.

e Degree received at age 16.

f Degree received at age 18.

**Table 3:** Multivariable logistic regression models^a^ reporting ORs and 95% CIs for reporting mental health dysfunction within the five questions^b^ comprising the mental health scale of the SF-36 survey, by specific potential demographic, cancer, social, and economic factors

	**Have you been a very nervous person? (question 9b)**			**Have you felt so down in the dumps that nothing could cheer you up? (question 9c)**			**Have you felt calm and peaceful? (question 9d)**			**Have you felt downhearted and blue? (question 9f)**			**Have you been a happy person? (question 9h)**		
**Characteristic**	**OR (95% CI)**	***P*****-value**	***P*****heterogeneity**^c^ (***P*****trend**^d^)	**OR (95% CI)**	***P*****-value**	***P*****heterogeneity**^c^ (***P*****trend**^d^)	**OR (95% CI)**	***P*****-value**	***P*****heterogeneity**^c^ (***P*****trend**^d^)	**OR (95% CI)**	***P*****-value**	***P*****heterogeneity**^c^ (***P*****trend**^d^)	**OR (95% CI)**	***P*****-value**	***P*****heterogeneity**^c^ (***P*****trend**^d^)
**Sex**															
Male	1.0			1.0			1.0			1.0			1.0		
Female	1.4 (1.3–1.6)	<0.001	<0.0001	1.7 (1.5–1.9)	<0.001	<0.0001	1.6 (1.5–1.8)	<0.001	<0.0001	1.6 (1.4–1.7)	<0.001	<0.0001	1.2 (1.1–1.4)	<0.001	<0.0001
**Diagnosis**															
Leukaemia	1.0			1.0			1.0			1.0			1.0		
Hodgkin lymphoma	1.1 (0.9–1.3)	0.433		1.0 (0.8–1.2)	0.732		1.1 (0.9–1.3)	0.468		1.0 (0.8–1.2)	0.617		1.3 (1.0–1.6)	0.021	
Non-Hodgkin lymphoma	1.0 (0.8–1.2)	0.863		1.1 (0.8–1.4)	0.620		1.2 (1.0–1.5)	0.086		0.9 (0.8–1.2)	0.615		1.4 (1.1–1.8)	0.003	
Central nervous system	1.3 (1.1–1.5)	0.001		1.2 (1.0–1.4)	0.036		1.2 (1.0–1.3)	0.024		1.1 (1.0–1.3)	0.080		1.4 (1.2–1.6)	<0.001	
Neuroblastoma	0.8 (0.6–1.1)	0.116		1.2 (0.9–1.6)	0.124		1.1 (0.9–1.3)	0.535		1.1 (0.9–1.4)	0.432		1.3 (1.0–1.7)	0.041	
Non-heritable retinoblastoma	1.1 (0.8–1.3)	0.884		1.0 (0.8–1.4)	0.737		1.1 (0.9–1.4)	0.373		1.1 (0.8–1.3)	0.681		1.4 (1.0–1.8)	0.024	
Heritable retinoblastoma	0.8 (0.6–1.1)	0.242		1.1 (0.8–1.5)	0.518		0.8 (0.6–1.1)	0.242		0.9 (0.7–1.2)	0.612		1.2 (0.9–1.6)	0.311	
Wilms	0.9 (0.8–1.1)	0.249		0.9 (0.8–1.2)	0.586		1.0 (0.9–1.2)	0.896		0.8 (0.7–1.0)	0.065		1.0 (0.8–1.2)	0.989	
Bone sarcoma	1.0 (0.8–1.3)	0.881		1.2 (0.9–1.6)	0.229		1.2 (1.0–1.6)	0.071		1.0 (0.8–1.3)	0.737		1.5 (1.1–1.9)	0.005	
Soft tissue sarcoma	0.9 (0.7–1.1)	0.398		1.0 (0.8–1.2)	0.853		1.1 (0.9–1.3)	0.491		1.0 (0.8–1.2)	0.877		1.2 (1.0–1.5)	0.085	
Other	0.9 (0.7–1.1)	0.174	0.0022	0.9 (0.8–1.1)	0.481	0.2762	0.9 (0.8–1.1)	0.379	0.1254	0.8 (0.7–1.0)	0.015	0.0273	1.0 (0.8–1.2)	0.898	0.0004
**Age at diagnosis**															
0–4 years	1.0			1.0			1.0			1.0			1.0		
5–9 years	1.1 (0.9–1.2)	0.412		1.0 (0.9–1.2)	0.527		1.0 (0.9–1.2)	0.423		1.1 (1.0–1.2)	0.200		1.0 (0.9–1.2)	0.618	
10–14 years	1.0 (0.9–1.2)	0.708	0.7132 (0.7301)	1.1 (0.9–1.3)	0.334	0.6190 (0.3569)	1.1 (1.0–1.3)	0.094	0.2444 (0.1029)	1.1 (1.0–1.3)	0.098	0.2266 (0.1072)	1.1 (1.0–1.3)	0.132	0.2990 (0.1519)
**Age at questionnaire completion**															
16–24 years	1.0			1.0			1.0			1.0			1.0		
25–34 years	1.1 (1.0–1.3)	0.131		1.2 (1.0–1.4)	0.210		1.2 (1.0–1.3)	0.010		1.3 (1.1–1.5)	<0.001		1.3 (1.1–1.5)	0.001	
35–44 years	1.0 (0.9–1.2)	0.852		1.2 (1.0–1.4)	0.103		1.3 (1.1–1.5)	0.002		1.3 (1.1–1.6)	0.001		1.5 (1.3–1.9)	<0.001	
45+ years	1.0 (0.8–1.2)	0.666	0.2051 (0.8380)	0.8 (0.6–1.0)	0.050	0.0001 (0.7357)	1.0 (0.8–1.3)	0.731	0.0021 (0.0415)	1.2 (1.0–1.5)	0.078	0.0013 (0.0015)	1.6 (1.2–2.0)	<0.001	<0.0001 (<0.0001)
**Marital status**															
Single	1.0			1.0			1.0			1.0			1.0		
Cohabiting	0.8 (0.7–1.0)	0.018		1.0 (0.8–1.2)	0.700		1.1 (1.0–1.3)	0.159		1.0 (0.8–1.2)	0.879		1.1 (0.9–1.3)	0.519	
Married	0.7 (0.6–0.8)	<0.001		0.6 (0.5–0.7)	<0.001		0.9 (0.8–1.0)	0.091		0.7 (0.6–0.8)	<0.001		0.7 (0.6–0.8)	<0.001	
Separated	1.2 (0.8–1.7)	0.305		1.3 (0.9–1.9)	0.149		1.3 (0.9–1.9)	0.098		1.7 (1.2–2.4)	0.003		1.3 (0.9–1.9)	0.175	
Divorced	1.0 (0.8–1.3)	0.916		1.1 (0.8–1.4)	0.588		1.0 (0.8–1.3)	0.822		1.0 (0.8–1.2)	0.872		1.1 (0.9–1.4)	0.469	
Widowed	0.9 (0.4–2.1)	0.810	<0.0001	2.1 (0.9–4.6)	0.074	<0.0001	1.3 (0.6–2.9)	0.455	0.0306	1.1 (0.5–2.3)	0.893	<0.0001	1.1 (0.5–2.5)	0.852	<0.0001
**Educational attainment**															
No qualifications	1.0			1.0			1.0			1.0			1.0		
Other qualifications	1.0 (0.9–1.2)	0.719		0.9 (0.7–1.0)	0.123		0.9 (0.7–1.0)	0.054		0.9 (0.8–1.1)	0.230		0.9 (0.7–1.1)	0.218	
O-level^e^	0.8 (0.7–0.9)	0.001		0.7 (0.6–0.8)	<0.001		0.8 (0.7–0.9)	0.004		0.8 (0.7–1.0)	0.013		0.8 (0.7–0.9)	0.010	
A-level^f^	0.7 (0.6–0.9)	<0.001		0.6 (0.5–0.8)	<0.001		0.8 (0.7–0.9)	0.004		0.7 (0.6–0.9)	<0.001		0.8 (0.6–0.9)	0.005	
Teaching qualification	0.8 (0.6–1.0)	0.029		0.6 (0.5–0.8)	<0.001		0.8 (0.7–1.0)	0.031		0.8 (0.6–0.9)	0.006		0.7 (0.6–0.9)	0.009	
Degree	0.6 (0.5–0.8)	<0.001	<0.0001	0.5 (0.4–0.6)	<0.001	<0.0001	0.8 (0.7–1.0)	0.047	0.0646	0.7 (0.6–0.8)	<0.001	<0.0001	0.7 (0.6–0.9)	0.002	0.0007
**SEC**															
Student	1.0			1.0			1.0			1.0			1.0		
Never worked/unemployed	1.3 (1.1–1.6)	0.008		2.6 (2.1–3.2)	<0.001		1.3 (1.1–1.6)	0.006		1.5 (1.2–1.8)	<0.001		1.8 (1.5–2.3)	<0.001	
Routine/manual	1.0 (0.9–1.2)	0.892		1.4 (1.2–1.7)	<0.001		1.1 (0.9–1.2)	0.417		1.3 (1.1–1.5)	0.004		1.2 (1.0–1.4)	0.091	
Intermediate	0.9 (0.7–1.0)	0.153		1.1 (0.9–1.4)	0.245		1.0 (0.9–1.2)	0.986		1.1 (0.9–1.3)	0.195		1.1 (0.9–1.3)	0.430	
Managerial/professional	0.8 (0.6–0.9)	0.009	<0.0001	1.0 (0.8–1.3)	0.766	<0.0001	1.0 (0.8–1.2)	0.816	0.0339	0.9 (0.8–1.1)	0.527	<0.0001	1.0 (0.8–1.2)	0.973	<0.0001

Abbreviations: CI=confidence interval; CNS=central nervous system; NHL=non-Hodgkin lymphoma; OR=odds ratio; SEC=socio-economic classification.

a Adjusted for age at diagnosis, diagnosis, sex, age at questionnaire completion, marital status, educational attainment, and SEC.

b To view full questions, please refer to Figure 1.

c The Pheterogeneity (two-sided) is from the likelihood ratio test for heterogeneity in the probability of reporting mental health dysfunction within this specific question, across different levels of the specified explanatory factor with adjustment for all other factors in the multivariable model. The threshold for statistical significant was 0.05.

d The *P*trend (two-sided) is from the test for trend, where the threshold for statistical significant was 0.05.

e Degree received at age 16.

f Degree received at age 18.

**Table 4:** Percentage of individuals and corresponding 95% CIs reporting mental health dysfunction among the general health population sample (OHLS) and childhood cancer survivors within the British Childhood Cancer Survivor Study, adjusting for sex and age, for each question^a^

**Question statement**	**Scale**	**General population sample**	**All survivors**	**Leukaemia**	**Hodgkin lymphoma**	**NHL**	**CNS**	**Neuroblastoma**	**NH-retinoblastoma**	**H-retinoblastoma**	**Wilms**	**Bone sarcoma**	**Soft tissue sarcoma**	**Other**
Cut down on the amount of time you spent on work or other activities? (question 5a)	RE	12.7 (12.0–13.4)	16.2 (15.0–17.4)	10.6 (7.8–13.5)	13.6 (9.4–17.7)	14.9 (10.5–19.2)	21.6 (19.2–24.0)	14.9 (10.2–19.7)	11.2 (6.7–15.7)	18.1 (11.4–24.8)	14.1 (9.4–18.8)	20.8 (15.7–25.8)	18.2 (13.8–22.7)	11.6 (8.7–14.6)
Accomplished less than you would like? (question 5b)	RE	21.6 (20.7–22.4)	22.7 (21.4–24.1)	19.0 (16.1–22.0)	18.9 (14.3–23.4)	24.2 (18.8–29.7)	28.4 (25.8–31.0)	16.1 (12.2–20.0)	19.4 (14.6–24.1)	27.9 (20.4–35.4)	21.5 (15.3–27.8)	26.0 (20.7–31.3)	22.2 (17.6–26.8)	19.1 (15.7–22.5)
Did work or other activities less carefully than usual? (question 5c)	RE	17.6 (16.8–18.3)	17.3 (16.0–18.5)	11.3 (8.4–14.1)	18.7 (13.8–23.6)	17.5 (12.9–22.1)	22.0 (19.6–24.5)	12.9 (9.1–16.8)	13.8 (8.8–18.8)	16.7 (10.1–23.3)	17.9 (12.0–23.8)	19.2 (14.4–24.0)	19.2 (14.6–23.7)	12.5 (9.5–15.5)
Has your physical health or emotional problems interfered with your normal social activities? (question 6)	SF	14.7 (14.0–15.5)	19.9 (18.6–21.2)	16.2 (13.4–19.0)	14.7 (10.4–18.9)	21.4 (16.3–26.5)	26.0 (23.5–28.4)	17.1 (10.5–23.7)	14.7 (9.6–19.8)	18.7 (12.9–24.4)	15.0 (10.5–19.6)	25.4 (20.2–30.6)	20.9 (16.2–25.6)	15.2 (11.9–18.6)
Has your health limited your social activities? (question 9j)	SF	13.2 (12.5–13.9)	23.1 (21.8–24.5)	16.0 (13.2–18.8)	14.8 (10.5–19.1)	23.3 (18.2–28.4)	35.5 (32.8–38.2)	14.2 (10.3–18.2)	16.9 (11.2–22.6)	19.9 (12.9–27.0)	21.2 (14.8–27.6)	29.1 (23.6–34.7)	20.9 (16.3–25.6)	16.7 (13.1–20.3)
Have you been a very nervous person? (question 9b)	MH	20.6 (19.8–21.5)	28.3 (26.9–29.7)	25.1 (21.6–28.6)	27.5 (22.2–32.8)	25.8 (20.7–30.9)	36.2 (33.5–38.9)	19.4 (14.0–24.7)	25.7 (19.7–31.8)	20.4 (14.7–26.0)	24.0 (18.2–29.8)	23.5 (19.1–28.0)	26.6 (21.7–31.5)	23.3 (19.8–26.9)
Have you felt so down in the dumps that nothing could cheer you up? (question 9c)	MH	18.3 (17.5–19.0)	22.8 (21.6–24.0)	22.3 (19.3–25.2)	18.9 (14.5–23.3)	22.6 (17.6–27.6)	28.2 (25.7–30.7)	22.3 (17.6–27.0)	19.3 (14.3–24.3)	22.5 (15.5–29.5)	20.5 (15.5–25.5)	25.2 (19.9–30.5)	20.9 (16.5–25.4)	17.4 (14.7–20.1)
Have you felt calm and peaceful? (question 9d)	MH	43.3 (42.3–44.4)	45.1 (43.6–46.6)	39.5 (35.6–43.5)	45.3 (40.1–50.5)	45.9 (40.2–51.7)	49.2 (46.4–52.0)	42.2 (33.8–50.6)	44.3 (38.1–50.5)	34.4 (28.2–40.5)	45.1 (38.6–51.5)	50.2 (44.3–56.1)	43.0 (37.8–48.1)	40.3 (36.2–44.4)
Have you felt downhearted and blue? (question 9f)	MH	28.3 (27.4–29.2)	35.2 (33.7–36.7)	29.5 (26.6–32.5)	30.7 (25.6–35.7)	31.1 (25.7–36.5)	42.7 (39.9–45.5)	29.4 (24.4–34.3)	32.6 (27.2–37.9)	30.3 (22.9–37.6)	29.3 (23.9–34.7)	35.9 (30.1–41.7)	34.4 (29.2–39.5)	28.6 (24.8–32.4)
Have you been a happy person? (question 9h)	MH	25.3 (24.4–26.2)	25.9 (24.5–27.3)	19.5 (16.8–22.3)	26.0 (21.0–30.8)	27.6 (22.1–33.1)	30.7 (28.1–33.3)	23.3 (16.0–30.6)	24.5 (18.4–30.5)	20.5 (14.6–26.5)	26.9 (21.0–32.8)	32.4 (26.7–38.2)	25.3 (20.4–30.2)	19.5 (16.7–22.3)

Abbreviations: CIs=confidence intervals; CNS=central nervous system; H=heritable; MH=mental health; NH=non-heritable; NHL=non-Hodgkin lymphoma; RE=role emotional; SF=social functioning.

a To view full questions, please refer to Figure 1.

**Table 5:** Percentage of individuals and corresponding 95% CIs reporting mental health dysfunction by attained age among the general health population sample (OHLS) and childhood cancer survivors within the British Childhood Cancer Survivor Study, adjusting for sex and age, for each question^a^

		**Age at SF-36 Completion**							
		**16–24**		**25–34**		**35–44**		**45+**	
**Question statement**	**Scale**	**General population sample**	**All survivors**	**General population sample**	**All survivors**	**General population sample**	**All survivors**	**General population sample**	**All survivors**
Cut down on the amount of time you spent on work or other activities? (question 5a)	RE	14.4 (12.5–16.3)	12.9 (11.6–14.2)	12.1 (10.7–13.4)	15.4 (14.2–16.7)	12.1 (10.7–13.5)	17.2 (15.4–18.9)	12.8 (11.6–13.9)	17.5 (14.6–20.3)
Accomplished less than you would like? (question 5b)	RE	22.6 (20.4–24.9)	19.0 (17.5–20.6)	20.6 (18.9–22.3)	20.8 (19.5–22.2)	21.6 (19.9–23.3)	23.6 (21.7–25.6)	21.8 (20.4–23.2)	24.9 (21.7–28.1)
Did work or other activities less carefully than usual? (question 5c)	RE	21.6 (19.4–23.8)	14.8 (13.5–16.2)	17.5 (16.0–19.1)	16.7 (15.4–17.9)	18.1 (16.5–19.7)	18.1 (16.3–19.9)	15.6 (14.3–16.8)	18.1 (15.1–21.0)
Has your physical health or emotional problems interfered with your normal social activities? (question 6)	SF	16.9 (14.9–18.9)	16.1 (14.7–17.5)	13.5 (12.1–15.0)	18.9 (17.6–20.2)	14.1 (12.6–15.5)	20.4 (18.5–22.2)	15.1 (13.9–16.3)	21.8 (18.7–24.9)
Has your health limited your social activities? (question 9j)	SF	11.2 (9.6–12.9)	15.7 (14.2–17.1)	11.2 (9.9–12.5)	20.5 (19.1–21.8)	13.4 (12.0–14.8)	22.9 (20.9–24.8)	15.3 (14.1–16.5)	28.1 (24.7–31.5)
Have you been a very nervous person? (question 9b)	MH	23.7 (21.4–25.9)	30.7 (28.9–32.5)	20.3 (18.6–21.9)	29.7 (28.1–31.2)	20.9 (19.2–22.6)	26.8 (24.8–28.8)	19.5 (18.2–20.8)	27.5 (24.2–30.8)
Have you felt so down in the dumps that nothing could cheer you up? (question 9c)	MH	21.2 (19.1–23.4)	25.3 (23.6–26.9)	17.6 (16.0–19.2)	25.7 (24.2–27.1)	18.1 (16.5–19.6)	24.5 (22.6–26.5)	17.6 (16.3–18.9)	18.7 (15.9–21.6)
Have you felt calm and peaceful? (question 9d)	MH	44.8 (42.2–47.5)	43.2 (41.3–45.1)	45.0 (42.9–47.0)	46.8 (45.1–48.4)	47.1 (45.1–49.2)	47.7 (45.4–49.9)	39.1 (37.5–40.8)	43.1 (39.4–46.7)
Have you felt downhearted and blue? (question 9f)	MH	31.1 (28.7–33.6)	35.1 (33.3–37.0)	29.4 (27.5–31.3)	36.4 (34.8–38.0)	28.4 (26.6–30.3)	35.7 (33.5–37.9)	26.4 (24.9–27.9)	34.1 (30.6–37.6)
Have you been a happy person? (question 9h)	MH	22.0 (19.8–24.2)	21.5 (19.9–23.1)	24.1 (22.3–25.9)	24.5 (23.1–26.0)	27.1 (25.2–28.9)	27.1 (25.0–29.1)	26.2 (24.7–27.7)	27.8 (24.5–31.1)

Abbreviations: CIs=confidence intervals; MH=mental health; RE=role emotional; SF=social functioning.

a To view full questions, please refer to Figure 1.
