# Supplementary material for: Aspects of mental health dysfunction among survivors of childhood cancer
Source: Br J Cancer. 2015 Sep 29;113(7):1121–32. doi: 10.1038/bjc.2015.310 (PMC4651126; doi:10.1038/bjc.2015.310)
Supplement: Supplementary Table 1 [file bjc2015310x1.docx]

|  | **ROLE EMOTIONAL SCALE** | | | | | | **SOCIAL FUNCTIONING SCALE** | | | | **MENTAL HEALTH SCALE** | | | | | | | | | |
| --- | --- | --- | --- | --- | --- | --- | --- | --- | --- | --- | --- | --- | --- | --- | --- | --- | --- | --- | --- | --- |
|  | **Cut down on the amount of time you spent on work or other activities?**  **(Question 5a)** | | **Accomplished less than you would like?**  **(Question 5b)** | | **Did work or other activities less carefully than ususal?**  **(Question 5c)** | | **Has your physical health or emotional problems interfered with your normal social activities?**  **(Question 6)** | | **Has your health limited your social activities?**  **(Question 9j)** | | **Have you been a very nervous person?**  **(Question 9b)** | | **Have you felt so down in the dumps that nothing could cheer you up?**  **(Question 9c)** | | **Have you felt calm and peaceful?**  **(Question 9d)** | | **Have you felt downhearted and blue?**  **(Question 9f)** | | **Have you been a happy person?**  **(Question 9h)** | |
| **Characteristic** | No. Reporting Dysfunction/No.  Responders (%) | | No. Reporting Dysfunction/No.  Responders (%) | | No. Reporting Dysfunction/No.  Responders (%) | | No. Reporting Dysfunction/No.  Responders (%) | | No. Reporting Dysfunction/No.  Responders (%) | | No. Reporting Dysfunction/No.  Responders (%) | | No. Reporting Dysfunction/No.  Responders (%) | | No. Reporting Dysfunction/No.  Responders (%) | | No. Reporting Dysfunction/No.  Responders (%) | | No. Reporting Dysfunction/No.  Responders (%) | |
| **Sex** |  |  |  |  |  |  |  |  |  |  |  |  |  |  |  |  |  |  |  |  |
| Male | 610/5222 (11.7) | | 903/5221 (17.3) | | 670/5212 (12.9) | | 796/5300 (15.0) | | 847/5249 (16.1) | | 1328/5271 (25.2) | | 1018/5279 (19.3) | | 2000/5278 (37.9) | | 1530/5258 (29.1) | | 1139/5274 (21.6) | |
| Female | 846/4999 (16.9) | | 1155/4996 (23.1) | | 934/4974 (18.8) | | 1022/5081 (20.1) | | 1075/5026 (21.4) | | 1626/5065 (32.1) | | 1425/5064 (28.1) | | 2527/5062 (49.9) | | 1975/5050 (39.1) | | 1283/5065 (25.3) | |
| **Diagnosis** |  |  |  |  |  |  |  |  |  |  |  |  |  |  |  |  |  |  |  |  |
| Leukemia | 321/2811 (11.4) | | 478/2812(17.0) | | 368/2801 (13.0) | | 410/2852 (14.4) | | 374/2821 (13.3) | | 848/2833 (29.9) | | 686/2842 (24.1) | | 1190/2836 (42.0) | | 977/2827 (34.6) | | 559/2836 (19.7) | |
| Hodgkin | 93/732 (12.7) | | 132/730 (18.1) | | 113/731 (15.5) | | 104/737 (14.1) | | 101/734 (13.8) | | 197/736 (26.8) | | 144/738 (19.5) | | 316/739 (42.8) | | 231/738 (31.3) | | 182/737 (24.7) | |
| NHL | 75/527 (14.2) | | 110/530 (20.8) | | 90/525 (17.1) | | 85/531 (16.0) | | 84/527 (15.9) | | 133/531 (25.1) | | 110/530 (20.8) | | 236/530 (44.5) | | 160/528 (30.3) | | 133/530 (25.1) | |
| CNS | 437/2153 (20.3) | | 585/2153 (27.2) | | 455/2145 (21.2) | | 575/2230 (25.8) | | 717/2190 (32.7) | | 771/2213 (34.8) | | 643/2212 (29.1) | | 1091/2216 (49.2) | | 887/2201 (40.5) | | 666/2219 (30.0) | |
| Neuroblastoma | 55/424 (13.0) | | 79/425 (18.6) | | 59/423 (14.0) | | 72/426 (16.9) | | 66/423 (15.6) | | 100/426 (23.5) | | 107/426 (25.1) | | 177/424 (41.8) | | 145/425 (34.1) | | 98/406(23.2) | |
| Non-Heritable Retinoblastoma | 47/407 (11.6) | | 69/405 (17.0) | | 51/405 (12.6) | | 53/407 (13.0) | | 53/404 (13.1) | | 109/406 (26.9) | | 86/406 (21.2) | | 174/406 (42.9) | | 132/406 (32.5) | | 94/406 (23.2) | |
| Heritable Retinoblastoma | 43/288 (14.9) | | 68/290 (23.5) | | 42/288 (14.6) | | 48/293 (16.4) | | 49/291 (16.8) | | 66/292 (22.6) | | 62/292 (21.2) | | 111/293 (37.9) | | 88/292 (30.1) | | 65/293 (22.2) | |
| Wilms | 119/939 (12.7) | | 153/935 (16.4) | | 132/934 (14.1) | | 139/945 (14.7) | | 129/935 (13.8) | | 244/941 (25.8) | | 197/941 (20.9) | | 396/941 (41.9) | | 276/940 (29.3) | | 186/938 (19.8) | |
| Bone Sarcoma | 75/404 (18.6) | | 97/403 (24.1) | | 74/401 (18.5) | | 100/410 (24.4) | | 114/407 (28.0) | | 107/409 (26.2) | | 96/409 (23.5) | | 199/410 (48.5) | | 139/408 (34.1) | | 113/410 (27.6) | |
| Soft Tissue Sarcoma | 100/697 (14.4) | | 138/696 (19.8) | | 120/696 (17.2) | | 117/702 (16.7) | | 122/701 (17.4) | | 179/703 (25.5) | | 145/703 (20.6) | | 299/701 (42.7) | | 229/699 (32.8) | | 156/701 (22.3) | |
| Other | 91/839(10.9) | | 149/838 (17.8) | | 100/837 (12.0) | | 115/848 (13.6) | | 113/842 (13.4) | | 200/846 (24.6) | | 167/844 (19.8) | | 338/844 (41.1) | | 241/844 (28.9) | | 170/844 (20.1) | |
| **Age at Diagnosis** |  |  |  |  |  |  |  |  |  |  |  |  |  |  |  |  |  |  |  |  |
| Mean (range) | 7.1(0-14.9) | | 7(0-14.9) | | 6.9(0-14.9) | | 7.0(0-14.9) | | 7.2(0-14.9) | | 6.5(0-14.9) | | 6.5(0-14.9) | | 6.8(0-14.9) | | 6.7(0-14.9) | | 7(0-14.9) | |
| 0-4 years | 595/4711 (12.6) | | 837/4707 (17.8) | | 687/4697 (14.6) | | 762/4781 (15.9) | | 768/4736 (16.2) | | 1361/4764 (28.6) | | 1136/4767 (23.8) | | 1983/4761 (41.7) | | 1555/4752 (32.7) | | 1024/4766 (21.5) | |
| 5-9 years | 403/2710 (14.9) | | 578/2708 (21.3) | | 427/2703 (15.8) | | 497/2754 (18.1) | | 537/2720 (19.7) | | 805/2731 (29.5) | | 644/2734 (23.6) | | 1216/2739 (44.4) | | 941/2723 (34.6) | | 646/2730 (23.7) | |
| 10-14 years | 458/2800 (16.4) | | 643/2802 (23.0) | | 490/2786 (17.6) | | 559/2846 (19.6) | | 617/2819 (21.9) | | 788/2841 (27.7) | | 663/2842 (23.3) | | 1328/2840 (46.8) | | 1009/2833 (35.6) | | 752/2843 (26.5) | |
| **Radiotherapy** |  |  |  |  |  |  |  |  |  |  |  |  |  |  |  |  |  |  |  |  |
| No | 313/2206 (14.2) | | 455/2210 (20.6) | | 336/2203 (15.3) | | 381/2233 (17.1) | | 430/2214 (19.4) | | 617/2232 (27.6) | | 534/2229 (24.0) | | 989/2227 (44.4) | | 770/2225 (34.6) | | 548/2226 (24.6) | |
| Yes | 782/5144 (15.2) | | 1079/5140 (21.0) | | 837/5119 (16.4) | | 999/5249 (19.0) | | 1060/5188 (20.4) | | 1500/5213 (28.8) | | 1263/5224 (24.2) | | 2329/5221 (44.6) | | 1828/5203 (35.1) | | 1255/5219 (24.1) | |
| **Chemotherapy** |  |  |  |  |  |  |  |  |  |  |  |  |  |  |  |  |  |  |  |  |
| No | 535/3249 (16.5) | | 745/3248 (22.9) | | 556/3240 (17.2) | | 674/3322 (20.3) | | 802/3279 (24.5) | | 941/3309 (28.4) | | 809/3305 (24.5) | | 1505/3303 (45.6) | | 1186/3295 (36.0) | | 873/3305 (26.4) | |
| Yes | 510/7122 (13.2) | | 722/3872 (18.7) | | 574/3854 (14.9) | | 638/3918 (16.3) | | 612/3886 (15.8) | | 1104/3901 (28.3) | | 921/3910 (23.6) | | 1684/3907 (43.1) | | 1315/3895 (33.8) | | 843/3901 (21.6) | |
| **Surgery** |  |  |  |  |  |  |  |  |  |  |  |  |  |  |  |  |  |  |  |  |
| No | 438/3353 (13.1) | | 625/3353 (18.6) | | 486/3340 (14.6) | | 864/4286 (20.2) | | 968/4235 (22.9) | | 980/3386 (28.9) | | 820/3393 (24.2) | | 1485/3390 (43.8) | | 1186/3383 (35.1) | | 1090/4268 (25.5) | |
| Yes | 679/4202 (16.2) | | 944/4200 (22.5) | | 713/4186 (17.0) | | 548/3402 (16.1) | | 546/3369 (16.2) | | 1198/4266 (28.1) | | 1018/4266 (23.9) | | 1928/4264 (45.2) | | 1478/4251 (34.8) | | 762/3384 (22.5) | |

eTable 1*:* Frequency of reporting mental health dysfunction in the ten questions^$^ under investigation among survivors of childhood cancer in the British Childhood Cancer Survivor Study

| **Age at Questionnaire Completion** |  |  |  |  |  |  |  |  |  |  |  |  |  |  |  |  |  |  |  |  |
| --- | --- | --- | --- | --- | --- | --- | --- | --- | --- | --- | --- | --- | --- | --- | --- | --- | --- | --- | --- | --- |
| Mean (range) | 31.7(16.0-66.1) | | 31.5(16.0-66.1) | | 31.0(16.0-66.1) | | 31.5(16.0-70.0) | | 32.4(16.0-7.0) | | 29.7(16.0-70.0) | | 29.9(16.0-66.1) | | 30.6(16.0-70.0) | | 30.6(16.0-66.1) | | 31.5(16.1-70.0) | |
| 16-24 years | 435/3704 (11.7) | | 643/3695 (17.4) | | 521/3693 (14.1) | | 549/3741 (14.7) | | 518/3705 (14.0) | | 1113/3727 (29.9) | | 864/3724 (23.2) | | 1505/3728 (40.4) | | 1182/3716 (31.8) | | 744/3726 (20.0) | |
| 25-34 years | 523/3531 (14.8) | | 714/3529 (20.2) | | 562/3519 (16.0) | | 657/3587 (18.3) | | 705/3552 (19.9) | | 1038/3567 (29.1) | | 894/3579 (25.0) | | 1626/3572 (45.5) | | 1265/3564 (35.5) | | 862/3572 (24.1) | |
| 35-44 years | 325/1976 (16.5) | | 456/1981 (23.0) | | 349/1973 (17.7) | | 400/2024 (19.8) | | 443/2008 (22.1) | | 533/2021 (26.4) | | 496/2023 (24.5) | | 956/2021 (47.3) | | 712/2013 (35.4) | | 538/2021 (26.6) | |
| 45+ years | 173/1010 (17.1) | | 245/1012 (24.2) | | 172/1001 (17.2) | | 212/1029 (20.6) | | 256/1010 (25.4) | | 270/1021 (26.4) | | 189/1017 (18.6) | | 440/1019 (43.2) | | 346/1015 (34.1) | | 278/1020 (27.3) | |
| **Marital Status** |  |  |  |  |  |  |  |  |  |  |  |  |  |  |  |  |  |  |  |  |
| Single | 785/5629 (14.0) | | 1125/5629 (20.0) | | 908/5619 (16.2) | | 1026/5741 (17.9) | | 1070/5674 (18.9) | | 1756/5705 (30.8) | | 1423/5717 (24.9) | | 2437/5712 (42.7) | | 1967/5695 (34.5) | | 1318/5715 (23.1) | |
| Cohabiting | 166/1034 (16.1) | | 229/1029 (22.3) | | 170/1029 (16.5) | | 192/1043 (18.4) | | 187/1036 (18.1) | | 264/1043 (25.3) | | 254/1043 (24.4) | | 492/1041 (47.3) | | 375/1042 (36.0) | | 269/1038 (25.9) | |
| Married | 338/2703 (12.5) | | 473/2702 (17.5) | | 345/2689 (12.8) | | 408/2723 (15.0) | | 451/2704 (16.7) | | 645/2720 (23.7) | | 500/2717 (18.4) | | 1172/2717 (43.1) | | 792/2711 (29.2) | | 561/2716 (20.7) | |
| Separated | 35/149 (23.5) | | 50/150 (33.3) | | 39/148 (26.4) | | 36/150 (24.0) | | 36/148 (24.3) | | 54/150 (36.0) | | 51/149 (34.2) | | 81/150 (54.0) | | 79/148 (53.4) | | 49/149 (32.9) | |
| Divorced | 86/440 (19.6) | | 118/442 (26.7) | | 93/438 (21.2) | | 104/447 (23.3) | | 112/442 (25.3) | | 145/444 (32.6) | | 132/441 (29.9) | | 217/444 (48.9) | | 182/444 (41.0) | | 144/445 (32.4) | |
| Widowed | 6/32 (18.8) | | 8/32 (25.0) | | 9/32 (28.1) | | 8/32 (25.0) | | 13/33 (39.4) | | 10/33 (30.3) | | 13/33(39.4) | | 17/33 (51.5) | | 13/32 (40.6) | | 11/33 (33.3) | |
| **Educational Attainment** |  | |  | |  | |  | |  | |  | |  | |  | |  | |  | |
| No qualifications | 328/1433 (22.9) | | 416/1434 (29.0) | | 334/1418 (23.6) | | 453/1518 (29.8) | | 530/1475 (35.9) | | 544/1491 (36.5) | | 505/1489 (33.9) | | 761/1493 (51.0) | | 635/1482 (42.9) | | 456/1491 (30.6) | |
| Other qualifications | 219/1424 (15.4) | | 297/1421 (20.9) | | 231/1414 (16.3) | | 289/1447 (20.0) | | 312/1432 (21.8) | | 4991435 (34.8) | | 417/1441 (28.9) | | 649/1439 (45.1) | | 541/1433 (37.8) | | 375/1440 (26.0) | |
| O-level^a^ | 357/2836 (12.6) | | 516/2839 (18.2) | | 419/2837 (14.8) | | 433/2858 (15.2) | | 467/2835 (16.5) | | 780/2860 (27.3) | | 662/2855 (23.2) | | 1204/2858 (42.1) | | 972/2851 (34.1) | | 635/2853 (22.3) | |
| A-level^b^ | 236/1919 (12.3) | | 358/1921 (18.6) | | 258/1917 (13.5) | | 274/1928 (14.2) | | 244/1916 (12.7) | | 493/1923 (25.6) | | 370/1928 (19.2) | | 786/1926 (40.8) | | 581/1922 (30.2) | | 400/1925 (20.8) | |
| Teaching qualification | 111/919 (12.1) | | 158/917 (17.2) | | 128/917 (14.0) | | 124/919 (13.5) | | 136/913 (14.9) | | 236/914 (25.8) | | 177/918 (19.3) | | 392/918 (42.7) | | 282/914 (30.9) | | 195/916 (21.3) | |
| Degree | 151/1416 (10.7) | | 248/1416 (17.5) | | 173/1416 (12.2) | | 176/1423 (12.4) | | 156/1415 (11.0) | | 299/1426 (21.0) | | 225/1423 (15.8) | | 610/1425 (42.8) | | 390/1420 (27.5) | | 290/1424(20.4) | |
| **Socioeconomic Classification (SEC)** |  | |  | |  | |  | |  | |  | |  | |  | |  | |  | |
| Student | 206/1728 (11.9) | | 315/1725 (18.3) | | 241/1720 (14.0) | | 249/1738 (14.3) | | 232/1725 (13.5) | | 524/1733 (30.2) | | 351/1733 (20.3) | | 679/1734 (39.2) | | 512/1724 (29.7) | | 313/1733 (18.1) | |
| Never worked/Unemployed | 130/576 (22.6) | | 179/579 (30.9) | | 125/576 (21.7) | | 174/603 (28.9) | | 191/600 (31.8) | | 231/60 (38.5) | | 244/604 (40.4) | | 295/603 (48.9) | | 252/601 (41.9) | | 203/601 (33.8) | |
| Routine/Manual | 435/3086 (14.1) | | 598/3080 (19.4) | | 501/3071 (16.3) | | 540/3121 (17.3) | | 589/3083 (19.1) | | 944/3105 (30.4) | | 799/3103 (25.8) | | 1353/3102 (43.6) | | 1134/3102 (36.6) | | 754/3106 (24.3) | |
| Intermediate | 253/1840 (13.8) | | 365/1842 (19.8) | | 264/1838(14.4) | | 307/1847 (16.6) | | 316/1834 (17.2) | | 485/1844(26.3) | | 388/1843 (21.1) | | 807/1846 (43.7) | | 621/1836 (33.8) | | 423/1843 (23) | |
| Managerial/Professional | 236/2344 (10.1) | | 376/2345 (16.0) | | 276/2343 (11.8) | | 279/2353 (11.9) | | 275/2334 (11.8) | | 500/2354 (21.2) | | 382/2352 (16.2) | | 985/2352 (41.9) | | 645/2346 (27.5) | | 478/2349 (20.4) | |

eTable 1 (*continued):* Frequency of reporting mental health dysfunction in the ten questions^$^ under investigation among survivors of childhood cancer in the British Childhood Cancer Survivor Study

^$^To view full questions, please refer to Figure 1

^a^Degree received at age 16

^b^Degree received at age 18
